# Transmission and characterization of *bla*_NDM-1_ in *Enterobacter cloacae* at a teaching hospital in Yunnan, China

**DOI:** 10.1186/s12941-017-0232-y

**Published:** 2017-08-22

**Authors:** Na Du, Shumin Liu, Min Niu, Yong Duan, Shuangmeng Zhang, Jing Yao, Jian Mao, Ran Chen, Yan Du

**Affiliations:** Department of Clinical Laboratory, the First Affiliated Hospital of Kunming Medical University, Yunnan Institute of Laboratory Diagnosis, Yunnan Key Laboratory of Laboratory Medicine, No. 295, Xichang Road, Wuhua, Kunming, Yunnan China

**Keywords:** *Enterobacter cloacae*, NDM-1, ST74, Integron, ISCR1

## Abstract

**Background:**

In recent years, New Delhi metallo-beta-lactamases 1 (*bla*_NDM-1_) has been reported with increasing frequency and become prevalent. The present study was undertaken to investigate the epidemiological dissemination of the *bla*_NDM-1_ gene in *Enterobacter cloacae* isolates at a teaching hospital in Yunnan, China.

**Methods:**

Antimicrobial susceptibility testing was performed using VITEK 2 system and E test gradient strips. The presence of integrons and insertion sequence common region 1 were examined by PCR and sequencing. Clonal relatedness was assessed by pulsed-field gel electrophoresis (PFGE) and multilocus sequence typing. Conjugation experiments and Southern blot hybridization were performed to determine the transferability of plasmids.

**Results:**

Ten *E. cloacae* isolates and their *Escherichia coli* transconjugants were exhibited similar resistant patterns to carbapenems, cephalosporins and penicillins. 8 (80%) of *E. cloacae* isolates carried class 1 integron and 1 (12.5%) carried class 2 integron. Integron variable regions harbored the genes which encoded resistance to aminoglycosides (*aadA1*, *aadA2*, *aadA5*, *aadB*, *aac(6′)*-*Ib*-*cr*), sulfamethoxazole/trimethoprim (*dfrA17*, *dfrA12*, *dfrA15*) and Streptozotocin (*sat2*). Six *E. cloacae* isolates belonged to ST74 and exhibited highly similar PFGE patterns. Each isolate shared an identical plasmid with ~33.3 kb size that carried the *bla*_NDM-1_ gene, except T3 strain, of which the *bla*_NDM-1_ gene was located on a ~50 kb plasmid.

**Conclusions:**

Our findings suggested that plasmid was able to contribute to the dissemination of *bla*_NDM-1_. Hence, more attention should be devoted to monitor the dissemination of the *bla*_NDM-1_ gene due to its horizontal transfer via plasmid. In addition, nosocomial surveillance system should actively monitor the potential endemic clone of ST74 to prevent their further spread.

## Background

*Enterobacter cloacae* is an important nosocomial pathogen, which can cause various infections including urinary tract, skin and soft tissue, respiratory tract, surgical site, biliary tract, sepsis, intravenous catheters, central nervous system and outbreaks at neonatal units [1, 2]. New Delhi metallo-beta-lactamases 1 (*bla*_NDM-1_) are Ambler class B Metallo-β-lactamases(MBLs)with carbapenemase activity that confers resistance to all β-lactams except aztreonam, was first identified in a carbapenem-resistant *Klebsiella pneumoniae* strain recovered from a Swedish patient who was hospitalized in India in 2008 [3], and mainly detected in carbapenem-resistant *Acinetobacter* spp. in mainland China. However, NDM-1-mediated carbapenem resistance in *E. cloacae* has been rarely reported in China.

The widespread dissemination of *bla*_NDM-1_ is mainly due to plasmids, integrons, insertion sequence common region (ISCR) and clonal outbreaks [4]. Plasmids are extrachromosomal DNA molecules capable of autonomous replication, and can confer resistance to the major antimicrobials [5]. Integrons are bacterial genetic elements able to capture and express genes contained within mobile gene cassettes. Typically, integrons are composed of two conserved regions, a 3′ conserved segment (3′CS) and a 5′ conserved segment (5′CS), as well as an internal variable region containing gene cassettes that encode antimicrobial resistance determinants [6]. ISCR elements can transpose adjacent DNA sequences by a process called rolling-circle replication, now it is recognized as powerful antibiotic resistance gene capture systems and playing a major role in spread of antibiotic resistance genes. However, whether these mobile elements mediate the dissemination of *bla*_NDM-1_ gene is still an unsolved mystery in the region. Therefore, the aim of this study is to explore the epidemiological dissemination of the *bla*_NDM-1_ gene in *E. cloacae* isolates at a teaching hospital in Yunnan, China.

## Methods

### Bacterial strains

In total, ten NDM-1-producing *E. cloacae* isolates were collected from hospitalized patients in the First Affiliated Hospital of Kunming Medical University between June 2012 and January 2016. All isolates were identified by VITEK2 automated identification system (bioMerieux, France). Carbapenemase activity was assessed by the modified Hodge test, MBL production was examined by imipenem-ethylenediaminetetraacetic acid (EDTA) disk method and the *bla*_NDM-1_ gene was determined by PCR in the early stage of the study. All patients were of Yunnan descent and none had a recent history of travelling epidemic area (Table 1).

**Table 1:** Clinical characteristics of the cases

Isolates	Age (years)	Gender	Specimen	Ward	Average hospital stay (days)	History of travel epidemic area	Outcome
T1	58	Male	Secreta	Orthopedics Department	66	No	Discharge
T2	96	Male	Sputum	General Practice	33	No	Discharge
T3	87	Female	Urine	EICU	117	No	Death
T4	36	Female	Urine	Transplantation Center	20	No	Discharge
T5	32	Male	Urine	Transplantation Center	39	No	Discharge
T6	65	Female	Pleural effusion	EICU	1	No	Death
T7	32	Male	Urine	Transplantation Center	23	No	Discharge
T8	22	Female	Urine	Transplantation Center	35	No	Discharge
T9	11	Female	Blood	NICU	18	No	Discharge
T10	34	Male	Urine	Transplantation Center	32	No	Discharge

*EICU* emergency intensive care unit, *NICU* neonatal intensive care unit

### Antimicrobial susceptibility testing

Antimicrobial susceptibilities for the NDM-1-producing isolates and transconjugants were initially tested using the VITEK2 system. MICs of imipenem, meropenem and ertapenem were re-evaluated using E test gradient strips (bioMerieux, France) on Mueller–Hinton agar plates and the results interpreted according to the CLSI guidelines [7]. *Escherichia coli* ATCC 25922 was used as quality control strain.

### PCR amplification and sequencing

Isolates were grown overnight in M–H Agar plates at 37 °C and genomic DNA was extracted using boiling method. Class 1, 2, 3 integrons and ISCR1 were, respectively, amplified using the primers intI1/intI1, intI2/intI2, intI3/intI3, hep58/hep59, hep74/hep51, orf513F/orf513R and orf513F/sul1R (Table 2). The amplified PCR products were analyzed by electrophoresis in 2% agarose gels and finally visualized in gel documentation system. PCR amplification products were sequenced. The resulting DNA sequences were analyzed by the BLAST program (http://www.ncbi.nlm.nih.gov/BLAST/).

**Table 2:** Primers used in PCR and DNA sequencing

Gene	Nucleotide sequence (5′ to 3′)	Expected size of amplicon (bp)	Reference or source
intI1/intI1	F1: GCATCCTCGGTTTTCTGG R1: GGTGTGGCGGGCTTCGTG	457	[8]
intI2/intI2	F2: CACGGATATGCGACA AAA AGGT R2: GTAGCA AACGAGTGACGA AATG	789	[8]
intI3/intI3	F3: ATCTGCCAA ACCTGACTG R3: CGA ATGCCCCAACAACTC	922	[8]
hep58/hep59	F4: TCATGGCTTGTTATGACTGT R4: GTAGGGCTTATTATGCACGC	Unknown	[9]
hep74/hep51	F5: CGGGATCCCGGACGGCATGCACGATTTGTA R5: GATGCCATCGCAAGTACGAG	Unknown	[10]
orf513F/orf513R	F6: ATGGTTTCATGCGGGTT R6: CTGAGGGTGTGAGCGAG	475	[11]
orf513F/sul1R	F7: ATGGTTTCATGCGGGTT R7: TTTGAAGGTTCGACAGC	Unknown	[11]

### Pulsed-field gel electrophoresis (PFGE) and multilocus sequence typing (MLST)

Bacterial genomic DNA was prepared in agarose plugs and digested with the restriction enzymes XbaI (Promega, USA). The DNA fragments were separated by use of a CHEF Mappar XA PFGE system (Bio-Rad, USA), with running time of 22 h and pulse times ranging from 5 to 40 s. The running buffer was 0.5 × Tris-boric acid-EDTA (TBE). *Salmonella ser. Braenderup H9812* was used as a standard for comparison. PFGE patterns were compared visually following previously described criteria [12]. Multilocus sequence typing (MLST) was performed on representative isolates. Internal fragments of the seven housekeeping genes were amplified using the primers given at the Institute Pasteur MLST Databases web site (http://pubmlst.org/ecloacae/). The PCR products were sequenced. Sequence types (STs) were assigned using online database tools.

### Conjugation experiments and Plasmid analysis

The transfer of carbapenem resistance was tested using a conjugation test, *E. coli* 600 (rifampicin-resistant) was used as the recipient strain, NDM-1-producing *E. cloacae* clinical isolates were used as the donor strains. Donor and recipient cells from M–H broth cultures were mixed in a ratio of 2:1 and transconjugant clones were screened on M–H agar plates containing rifampicin (256 mg/L) and Imipenem (1 mg/L). Conjugation events occurred at 37 °C. The presence of the *bla*_NDM-1_ gene in transconjugants was determined by PCR and sequencing. Genomic DNA was digested with S1 nuclease (Promega, USA). The linearized plasmids and partially digested genomic DNA were separated through the CHEF-Mapper XA PFGE system with a switch time from 2.16 to 63.8 s for 18 h at 6 V/cm at 14 °C. Linear plasmids generated by S1-PFGE were transferred to nvlon membrane (Millipore, USA) and hybridized with a digoxigenin-labeled probe specific to *bla*_NMD-1_. Probe labeling and signal detection were carried out with DIG high primer DNA labeling and detection starter kit according to the manufacturer’s instructions (Roche Applied Sciences, Germany).

## Results

### Antimicrobial susceptibility testing

The antibiotic susceptibility results showed that all the NDM-1-producing *E. cloacae* isolates exhibited resistance to carbapenems, cephalosporins and penicillins. Only one isolates remained susceptible to aztreonam, which was not hydrolysed by metallo-carbapenemases, thus suggesting the presence of additional β-lactamases in the remaining isolates. Ten isolates exhibited different level resistance to tetracycline, amikacin, ciprofloxacin and tigecycline, seven isolates were resistance to tetracycline, one to amikacin, nine to ciprofloxacin and one to tigecycline. These results are summarized in Table 3.

**Table 3:** Antimicrobial drug susceptibility profiles (MICs in mg/L) for clinical isolates and transconjugants

Isolate no.	VITEK2										E test		
	PIP	TCY	ATM	CAZ	CIP	AMK	MEM	IMP	ETP	TGC	MEM	IMP	ETP
T1	≥128	≥16	≥64	≥64	≥4	16	8	8	≥8	1	>32	32	>32
T2	≥128	4	≥64	≥64	≥4	≤2	≥16	≥16	≥8	1	>32	>32	>32
T3	≥128	≥16	≥64	≥64	≥4	≥64	8	≥16	≥8	≥8	>32	>32	>32
T4	≥128	8	≥64	≥64	≥4	4	≥16	≥16	≥8	1	>32	24	>32
T5	≥128	≥16	16	≥64	≥4	4	≥16	≥16	≥8	2	>32	>32	>32
T6	≥128	≥16	≥64	≥64	≥4	16	≥16	≥16	≥8	1	32	>32	>32
T7	≥128	≥16	16	≥64	≥4	16	≥16	≥16	≥8	2	>32	>32	>32
T8	≥128	≥16	≥64	≥64	≥4	16	≥16	≥16	≥8	1	>32	>32	>32
T9	≥128	≥16	≤1	≥64	0.5	≤2	≥16	≥16	≥8	1	>32	>32	>32
T10	≥128	4	≥64	≥64	≥4	≤2	4	4	≥8	2	6	8	8
T1-EC600	≥128	≤1	≤1	≥64	0.5	≤2	8	≥16	≥8	1	8	8	8
T2-EC600	≥128	≤1	32	≥64	0.5	≤2	≥16	≥16	≥8	1	8	16	>32
T3-EC600	≥128	≤1	≤1	≥64	0.5	≤2	8	≥16	≥8	2	16	16	>32
T4-EC600	≥128	≥16	32	≥64	0.5	≤2	8	≥16	≥8	1	>32	6	8
T5-EC600	≥128	≤1	≤1	≥64	0.5	≤2	8	≥16	≥8	2	>32	>32	>32
T6-EC600	≥128	≤1	≤1	≥64	0.5	≤2	≥16	≥16	≥8	1	>32	>32	>32
T7-EC600	≥128	≤1	≤1	≥64	0.5	≤2	≥16	≥16	≥8	1	24	8	8
T8-EC600	≥128	≤1	≤1	≥64	0.5	≤2	8	≥16	≥8	1	>32	>32	>32
T9-EC600	≥128	≤1	≤1	≥64	0.5	≤2	≥16	≥16	≥8	1	24	>32	24
T10-EC600	≥128	≤1	≤1	≥64	0.5	≤2	≥16	≥16	≥8	1	>32	>32	16
EC600	≤4	≤1	≤1	≤1	≤0.25	≤2	≤0.25	≤1	≤0.5	≤0.5	0.032	0.019	0.008
ATCC25922	≤4	≤1	≤1	≤1	≤0.25	≤2	≤0.25	≤1	≤0.5	≤0.5	0.032	0.019	0.008

*EC600 Escherichia coli* 600, *T1*-*EC600* the transconjugants of T1 strain, *PIP* piperacillin, *TCY* tetracycline, *ATM* aztreonam, *CAZ* ceftazidime, *CIP* ciprofloxacin, *AMK* amikacin, *MEM* meropenem, *IPM* imipenem, *ETP* ertapenem, *TGC* tigecycline

### Detection of integrons and ISCR1

Class 1 integrase gene was detected in 80% (8/10), while the variable region of class 1 integron was detected in 70% (7/10) NDM-1-producing *E. cloacae* isolates. Among them, six different gene cassette arrays were found, which included: *dfrA12*+*aadA2*, *dfrA15*+*dfrA17*, *aadA2*+*aadA5*+*dfrA17*, *dfrA15*+*aadB*+*aadA2*, *dfrA15* and *dfrA15*+*aac (6′)*-*Ib*-*cr*. Those genes encoded resistance to aminoglycosides and sulfamethoxazole/trimethoprim. 10% (1/10) strains possessed class 2 integron, and the variable region of class 2 integron harbored *sat2*+*aadA1* genes, which mediated antibiotic resistance to streptothricin and streptomycin. None of the isolates harbored class 3 integron. 50% (5/10) isolates carried ISCR1 elements. However, in the five ISCR1 positive isolates, the cassette arrays could not be detected.

### PFGE and MLST typing

**Fig. 1:**
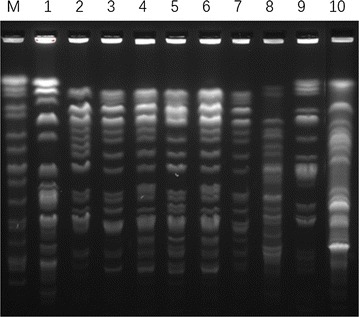
Pulsed-field gel electrophoresis (PFGE) of XbaI-digested DNA of *E. cloacae* isolates. *Lane M* PFGE marker, *Salmonella ser. Braenderup* H9812; *lanes 1*–*10* representative NDM-1-producing *E. cloacae*

According to Tenover’s criteria [12], Six *E. cloacae* isolates, of which four obtained from transplantation center, one from general practice and one from emergency intensive care unit, belonged to ST74 and shared the same PFGE fingerprint pattern (Fig. 1), suggesting they were clonally related, the remaining strains were characterized by unique genotypes. MLST of representative isolates assigned the *E. cloacae* to four sequence type (ST), ST74, ST182, ST754 and ST175, respectively.

### Plasmid analysis

**Fig. 2:**
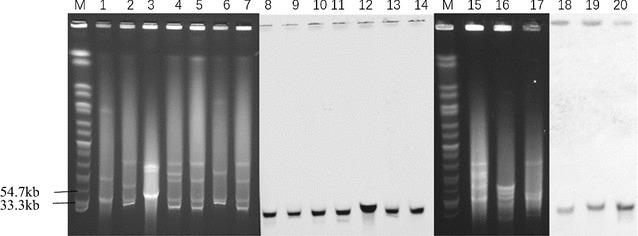
Results of S1 nuclease PFGE and Southern blot hybridization. *Lane M* PFGE marker, *Salmonella ser. Braenderup* H9812; *lanes 1*–*7* Strains (T1–T7) digested with S1 nuclease; *lanes 8*–*14* hybridized plasmids of strains (T7–T1) with the *bla*_NDM-1_ probe; *lanes 15*–*17* strains (T8–T10) digested with S1 nuclease; *lanes 18–20* hybridized plasmids of strains (T8–T10) with the *bla*_NDM-1_ probe

Conjugation experiments revealed that plasmids harboring *bla*_NDM-1_ were transformed into *E. coli* 600. All transconjugants conferred resistance to carbapenems, cephalosporins and penicillins while all of them remained susceptible to ciprofloxacin and amikacin (Table 1). Isolates harbored three to five plasmids according to S1-PFGE electrophoresis. Southern hybridization analysis with a *bla*_NDM-1_-specific probe revealed that *bla*_NDM-1_ was located on a ~33.3 kb plasmid in nine isolates and on a ~50 kb plasmid in one isolates (Fig. 2).

## Discussion

The *bla*_NDM-1_ was first identified in a clinical isolate of *K. pneumoniae* in New Delhi, India, and suddenly got disseminated around the world. The *bla*_NDM-1_-carrying bacteria conferred resistance to all most β-lactam antibiotics. Thus, treatment of infections with NDM-producing bacteria was a major challenge, leaving few options for clinical treatment beside tigecycline or colistin. The *bla*_NDM-1_ gene have been identified in a variety of gram-negative bacilli, including *Acinetobacter* spp. [13], *Enterobacteriaceae* [14] and *Pseudomonas aeruginosa* [15]. Lack of uncontaminated potable water and abuse of over-the-counter antibiotic administration offered the ideal setting for the development of a latent endemic situation, and international “medical tourism” played an important role in the spread of *bla*_NDM-1_ gene [16]. Yunnan Kunming is a famous tourist city in the world, and large numbers of domestic and foreign tourists come here every year, thus “medical tourism” may play an important role in the spread of *bla*_NDM-1_ among carbapenem-resistant bacteria in this region.

In China, plasmid-carrying bla_NDM-1_ have been identified in *Enterobacteriaceae* isolates in several regions including Beijing, Shanghai, Hong Kong, Shandong Henan and Yunnan province, the size of plasmids harboring *bla*_NDM-1_ were vary from ~50 to 360 kb [17, 18]. Our research shows that *bla*_NDM-1_ gene was mainly located on a plasmid with ~33.3 kb size, were different from previously reported in China before. The *bla*_NDM-1_ gene was not detected in integrons and ISCR1 mobile elements. Integrons only carried these genes encoding resistance to aminoglycosides [*aadA1*, *aadA2*, *aadA5*, *aadB*, *aac(6′)*-*Ib*-*cr*], sulfamethoxazole/trimethoprim (*dfrA17*, *dfrA12*, *dfrA15*) and Streptozotocin (*sat2*). Those results suggested that plasmid was the most important mobile element that mediated the dissemination of *bla*_NDM-1_ and integrons were the basis for the formation of multiple drug-resistant bacteria in the region. In addition, it was worth noting that there had been some evidences which demonstrated that integrons and ISCR mobile elements were located on plasmids [4], thus, the resistance gene through the plasmids spreading was not a single element, but by 1 + 1 or 1 + 2 mode. This mode of dissemination should arouse high concern of the relevant departments.

Clonal spread was an important factor involved in the prevalence of NDM-1-producing *Enterobacteriaceae*. Outbreak of NDM-1-producing *K. pneumoniae* ST105 and ST147 have been reported in Yunnan [17] and Xi’an [19], and NDM-1-producing *E. cloacae* ST120 have been reported in Henan [18], China, respectively. Our study showed that 5 clusters for 10 strains, 1 cluster from 6 closely related isolates was found to exhibit similarities which is more than 90%. The result suggested that 6 NDM-1-producing *E. cloacae* isolates were clonally related. However, there was no significant epidemiological relatedness among them. Hence, we could not trace their origin. MLST analysis revealed four ST types including ST74, ST754, ST175 and ST182, among them, ST74 was the major type in NDM-1-producing *E. cloacae* isolates, which was different from previous types reported before such as ST66, ST78, ST108, ST114 and ST120 [18]. ST74 has been identified among non-susceptible to ertapenem of *E. cloacae* isolates in North-Eastern France, and was associated with OXA-48-producing *E. cloacae* isolates in Spain [20, 21]. The present study is the first to report on an outbreak of NDM-1-producing *E. cloacae* ST74.

Infection control measures were strengthened to prevent the further transmission of *bla*_NDM-1_. They included hand hygiene, contact isolation, active screening, environmental surface disinfection, standard aseptic manipulation techniques, among them, hand hygiene was the most effective and economical strategy for reducing cross infection [16].

To conclude, our study demonstrated that plasmids were the most important elements that mediate horizontal transfer of the *bla*_NDM-1_ gene. Furthermore, we identified a potential endemic clone of ST74. The emergence of NDM-1-producing *E. cloacae* ST74 isolates is worrying, nosocomial surveillance system should pay more attention to prevent their further spread.

## Abbreviations

NDM-1: New Delhi metallo-beta-lactamase 1
ISCR1: insertion sequence common region 1
PCR: polymerase chain reaction
PFGE: pulse field gel electrophoresis
MLST: multilocus sequence typing
MBLs: metallo-β-lactamases
5CS & 3CS: 5′ & 3′-conserved segments
CLSI: Clinical and Laboratory Standards Institute
M–H: Mueller–Hinton
MIC: minimum inhibitory concentration
